# Vortioxetine’s impact on the autonomic nervous system in depressed children and adolescents: analysis of the heart rate variability

**DOI:** 10.1038/s41598-024-65278-9

**Published:** 2024-06-23

**Authors:** Michaela Krivosova, Peter Hutka, Igor Ondrejka, Zuzana Visnovcova, Dana Funakova, Igor Hrtanek, Nikola Ferencova, Zuzana Mlyncekova, Veronika Kovacova, Andrea Macejova, Tomas Kukucka, Juraj Mokry, Ingrid Tonhajzerova

**Affiliations:** 1 Jessenius Faculty of Medicine in Martin, Biomedical Centre Martin, Comenius University Bratislava, Martin, Slovakia; 2 Jessenius Faculty of Medicine in Martin, Psychiatric Clinic, Comenius University Bratislava, University Hospital Martin, Martin, Slovakia; 3 Department of Pharmacology, Jessenius Faculty of Medicine in Martin, Comenius University Bratislava, Martin, Slovakia; 4 Department of Physiology, Jessenius Faculty of Medicine in Martin, Comenius University Bratislava, Martin, Slovakia

**Keywords:** Human behaviour, Limbic system, Prefrontal cortex, Emotion, Neuroscience, Peripheral nervous system, Autonomic nervous system

## Abstract

Relationship between depressive disorder and autonomic nervous system has been already discussed. Reduced emotional regulation is supposed to be associated with prefrontal hypofunction and subcortical hyperactivity. The aim of this study was to determine the effect of vortioxetine on heart rate variability (HRV), a parameter of cardiac autonomic regulation, in depressed hospitalized paediatric patients and assess the clinical effectiveness of the drug in this population. We performed repeated polysomnography analyses at admission and after a short treatment in hospital (15.2 days on average) and measured various HRV parameters (RRi, pNN50, RMSSD, LF-HRV, HF-HRV) during wakefulness, N3 and REM sleep stages. Out of 27 study subjects, 67% have improved depression symptoms as well as anxiety and subjective sleep quality after short vortioxetine treatment. We have found a significant decrease in parasympathetic parameters pNN50, RMSSD and HF-HRV during N3 sleep phase, though not exclusively among vortioxetine responders. The anticipated increase in cardiovagal regulation after vortioxetine treatment was not demonstrated in this pilot study, possibly due to the drug’s multimodal mechanism and impact on the nucleus tractus solitarii, particularly its antagonism on 5HT-3 receptors. Application of selective drugs could further explain the effect of vortioxetine on HRV in depressed patients.

## Introduction

Mastering the capacity to adapt one’s emotional responses to match the specific context, referred to as emotion regulation, stands as a fundamental developmental milestone during early childhood and adolescence. These processes are underpinned by the autonomic nervous system (ANS), a central physiological network responsible for dynamically harmonizing real-time sensory inputs with internal motivational and goal-oriented^1^. The circuitry between ANS and central nervous system (CNS), shaped early in the course of the life, forms the basis for the adaptive neurovisceral regulation throughout one’s lifetime. Therefore, adolescence contributes significantly to mental well-being rendering this age group notably susceptible to mental disorders. Indeed, it is estimated that globally 14% of 10–19 aged children and adolescents experience a mental disorder. Major depressive disorder (MDD) is believed to affect approximately 1.1% of individuals in the 10–14-year-old group and 2.8% of those aged 15–19^2^.

The autonomic imbalance is associated with a lack of physiological adaptability, dynamic flexibility, and health. There are two main theories that support the idea that cardiac vagal regulation indexed by heart rate variability (HRV) is associated with autonomic and emotional regulation. First, polyvagal theory^3,4^ specifies that the cardiac-linked vagal efferent modulation from the nucleus ambiguus is connected with cranial nerves involved in the emotional expression and vocalization important for emotional and affective regulation in people. Thus, vagally-mediated HRV index (high frequency, HF-HRV) could be considered as an index of emotional regulation. Further, Thayer and Lane^5^ reported the significance of cardiac autonomic regulation in the context of neurovisceral integration model (NIM). It is based on the existence of the central autonomic network (CAN) as functional cortico-subcortical neural circuits linking the heart to the prefrontal cortex (PFC). More specifically, the CAN described by Benarroch^6^ includes several brain structures such as PFC, amygdala, specific medullary areas, which are important for the complex heart rate (HR) control characterizing a healthy and adaptive regulatory system^5^. PFC is an executive brain region responsible for emotion regulation, decision-making, and social behaviour. It exerts an inhibitory influence on subcortical centres including the amygdala that allows the organism to adjust emotional and behavioural responses. Amygdala, important part of the limbic system*,* controls predominantly emotional regulation and basic autonomic arousal processes^7^*.* More specifically, amygdala, activated during threat/uncertainty, is under tonic inhibitory control of the PFC. Thus, in case of threat or novelty associated with emotional regulation, the PFC becomes hypoactive. This hypoactive state is related to inhibition of cardiovagal regulatory mechanisms accompanied by an increase in HR and decrease of vagally mediated HRV^8–10^. Therefore, the PFC and its abnormalities (e.g. prefrontal hypoactivity) could play a critical role in vagally-mediated dysregulation associated with MDD because of the PFC disruption to inhibit the amygdala as a region that mediates cardiovascular and autonomic responses^11^.

The relationship between autonomic dysfunction and emotion dysregulation (ED) has been described previously in the literature: ADHD in childhood was associated with abnormal parasympathetic mechanisms^12^, borderline personality disorder (BPD) symptom severity was significantly related to reduced resting-state HRV and increased HR^13^, loss of HRV complexity (a novel feature to detect cardiac abnormalities) has been associated with psychopathologies such as schizophrenia, bipolar disorder, and MDD^10,11^.

Latest meta-analysis on MDD and HRV that included 21 studies found lower HRV-measures in MDD compared to healthy controls^14^. Specifically, patients with MDD had lower HF-HRV, lower percentage of successive R-R intervals that differ by more than 50 ms (pNN50), increased low-frequency (LF)/HF ratio, which suggests lower parasympathetic effect, thus impaired adaptability to the stimuli. The effect of antidepressants on HRV was observed in smaller number of studies and showed a great variability based on the treatment choice, duration of intervention, and HRV measurement characteristics. Usually, treatment with tricyclic antidepressants showed reduction in HRV, which could be explained by their anticholinergic properties^15,16^ while studies with selective serotonin reuptake inhibitors (SSRIs) were more variable—with no or negative effect on HRV^15–17^. In adolescents, one study found that at 8-week follow-up of SSRI antidepressants, HRV parameters increased in depressed patients, which correlated with the improved MDD symptoms^18^. HRV parameters were used also to predict treatment response in MDD^19^. Interestingly, psychotherapy was effective for improving reduced HRV in MDD patients^17^.

Vortioxetine is one of the newest antidepressants that, in comparison to SSRIs, not only block serotonin reuptake but also directly modulates various serotonin (5-HT) receptors: it is a 5-HT_3_, 5-HT_1D_, and 5-HT_7_ antagonist, a 5-HT_1A_ agonist, and a 5-HT_1B_ partial agonist^20^. It has been approved for the treatment of MDD in adults but has been studied also in paediatric population^21–23^.

Measurement of HRV during daytime can be influenced by many confounding factors such as level of stress, emotions, exercise, body posture, alcohol of caffeine intake, and others. Therefore, measurement in resting states such as during sleep minimizes the confounders^24^. However, HRV can vary also between different sleep stages given the changes in ANS activity. Deep non-REM sleep (stage N3) is a state with the most profound vagal influence on HR. Subsequently, transition to REM sleep causes a decrease in vagal contribution to HR control and results in a relative prevalence of low-frequency HRV^25^.

The primary goal of this study was to establish the influence of vortioxetine on HRV during individual sleep stages using polysomnography (PSG) analysis in hospitalized children and adolescents with the diagnosis of MDD. The objective is to correlate a determined effect of vortioxetine with previous literature findings on SSRIs based on polyvagal theory and NIM. Simultaneously, we aimed to describe the effectiveness of antidepressant treatment with vortioxetine in these patients.

## Results

**Figure 1:**
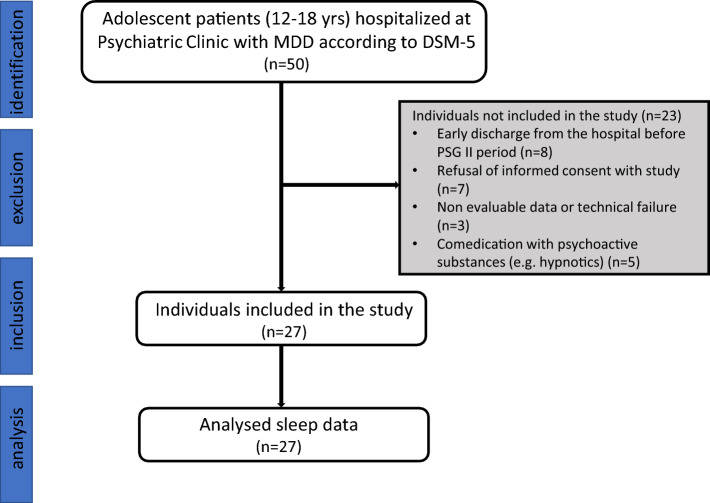
STROBE flow chart of participant selection procedure.

Out of 30 patients, the data from both PSG assessments were successfully obtained only from 27 patients as shown in Fig. 1. Their basic demographic data are as following: average age 15.1 ± 1.5 years old, 20 (74%) were girls and median vortioxetine daily dose was 10 mg. Psychiatric rating scale scores at admission (Time 1) and discharge from hospital (Time 2) are displayed in Table 1 and divided into group of responders and non-responders. In all measured rating scales, there is a significant improvement in responder group.

**Table 1:** Psychiatric rating scale scores before and after short vortioxetine treatment in hospital.

	Responders (N = 18)					Non-responders (N = 9)				
	Time 1 ± SD	RANGE	Time 2 ± SD	RANGE	*p-value*	Time 1 ± SD	RANGE	Time 2 ± SD	RANGE	*p-value*
MADRS	30.6 ± 10.0	34	7.3 ± 3.6	12	** < *0.0001***	23.9 ± 9.6	33	17.8 ± 5.0	15	*0.0729*
HAM-A	18.2 ± 8.5	31	6.4 ± 4.8	19	** < *0.0001***	12.9 ± 4.9	15	10.0 ± 3.3	9	*0.1204*
AIS	8.0 ± 6.3	20	3.8 ± 3.1	11	***0.0026***	7.8 ± 5.3	17	6.9 ± 5.0	14	*0.4219*
CDI A	5.0 ± 2.8	10	1.9 ± 1.6	5	** < *0.0001***	6.2 ± 2.7	7	5.9 ± 2.8	8	*0.7153*
CDI B	2.1 ± 1.2	5	0.7 ± 0.8	2	***0.0005***	2.0 ± 1.9	6	2.1 ± 1.2	4	*0.9688*
CDI C	3.9 ± 2.3	7	2.1 ± 1.7	5	***0.0001***	3.9 ± 1.9	6	4.4 ± 1.3	4	*0.4161*
CDI D	6.2 ± 3.0	11	3.7 ± 2.3	9	***0.0016***	6.3 ± 4.6	13	6.3 ± 4.2	13	> *0.9999*
CDI E	4.3 ± 2.7	9	2.0 ± 1.8	6	** < *0.0001***	4.7 ± 2.4	8	5.0 ± 3.0	8	*0.4379*
CDI total	21.6 ± 10.8	39	10.4 ± 6.3	22	** < *0.0001***	23.1 ± 12.1	35	23.8 ± 11.0	34	*0.7266*

MADRS—Montgomery-Asberg depression rating scale, HAM-A—Hamilton anxiety rating scale, AIS—Athens insomnia scale, CDI—child depression inventory: A—negative mood, B—interpersonal problems, C—ineffectiveness, D—anhedonia, E—negative self-esteem.

Significant p-values are in bolditalics and italics.

We have found a significant decrease in parameters pNN50 (*p* = 0.0169), RMSSD (*p* = 0.0084), and HF-HRV (*p* = 0.0178) during deep sleep (N3 sleep stage) after short vortioxetine treatment. The effect size of these results was moderate.

Comparing separately the data only in the responder group (N = 18), there were no significant differences in HRV parameters before and after short vortioxetine therapy. However, we have observed a significant decrease of the parameter RMSSD (*p* = 0.0369, d = 0.42) during N3 sleep stage and interestingly, a significant decrease of HF (*p* = 0.0443, d = 0.45) during REM sleep in the non-responder group (N = 9). All results and values of HRV parameters in various sleep stages and wakefulness in the study subjects in Time 1 and Time 2 are displayed in Table 2.

**Table 2:** HRV parameters in all subjects, responders, and non-responders before and after short vortioxetine treatment in hospital: RMSSD, HF, LF, RRi, pNN50.

	RMSSD								
	All subjects (N = 27)			Responders (N = 18)			Non-responders (N = 9)		
	Wake	N3	REM	Wake	N3	REM	Wake	N3	REM
Time 1									
Mean	64.4	70.4	63.2	54.5	63.7	58.8	84.2	83.8	71.9
SD	39.3	45.0	48.3	33.7	43.5	48.4	44.2	47.6	49.6
SEM	7.6	8.7	9.3	8.0	10.3	11.4	14.7	15.9	16.5
Range	158.5	158.6	187.6	136.6	139.7	187.6	135.2	149.2	144.8
Time 2									
Mean	58.6	53.5	52.7	51.5	47.9	50.0	72.8	64.8	58.1
SD	29.6	31.7	43.7	22.6	28.0	49.2	37.7	37.3	31.9
SEM	5.7	6.1	8.4	5.3	6.6	11.6	12.6	12.4	10.6
Range	123.4	126.2	188.5	79.4	91.8	188.5	116.6	115.0	98.5
dF	1			1			1		
F	9.414			3.575			8.777		
*p-value* ANOVA	**0.0030**			0.0643			**0.0068**		
% of total variation	1.904			1.443			3.285		
*p-value* Time 1 versus Time 2	0.3562	**0.0084**	0.0970	0.7237	0.0667	0.3006	0.1993	**0.0369**	0.1224
Cohen’s d	0.16	**0.42**	0.24	0.10	**0.41**	0.18	0.20	**0.42**	0.25

	HF								
	All subjects (N = 27)			Responders (N = 18)			Non-responders (N = 9)		
	Wake	N3	REM	Wake	N3	REM	Wake	N3	REM
Time 1									
Mean	1839	2340	1995	1274	2019	1746	2968	2981	2492
SD	2352	2567	2941	1645	2434	3006	3175	2850	2916
SEM	452.7	494.0	566.1	387.8	573.8	708.5	1058	950	972
Range	10,507	8671	11,841	7147	7879	11,841	10,036	8553	8105
Time 2									
Mean	1491	1346	1542	1043	1092	1648	2388	1853	1330
SD	1750	1530	2763	758.6	1089	3288	2716	2158	1327
SEM	336.9	294.5	531.7	178.8	256.7	775.0	905	719	442
Range	8597	7009	13,156	2451	3205	13,156	8573	6891	4145
dF	1			1			1		
F	6.368			1.710			9.161		
*p-value* ANOVA	**0.0137**			0.1969			**0.0058**		
% of total variation	1.614			0.8963			3.606		
*p-value* Time 1 versus Time 2	0.4002	**0.0178**	0.2734	0.6790	0.1007	0.8603	0.3000	0.0503	**0.0443**
Cohen’s d	0.16	**0.44**	0.16	0.16	**0.44**	0.03	0.12	**0.42**	**0.45**

	LF								
	All subjects (N = 27)			Responders (N = 18)			Non-responders (N = 9)		
	Wake	N3	REM	Wake	N3	REM	Wake	N3	REM
Time 1									
Mean	1617	935	1690	1260	1029	1501	2331	748	2067
SD	1820	1432	1809	1199	1659	1597	2617	870	2230
SEM	350.3	275.6	348.1	282.7	391.0	376.5	872	290	743
Range	7028	6330	6894	4837	6319	6894	7017	2510	6377
Time 2									
Mean	1569	940	1557	1273	733	1395	2161	1355	1882
SD	1210	1373	2004	798.1	1215	1958	1677	1643	2176
SEM	232.8	264.3	385.8	188.1	286.4	461.4	559	548	725
Range	5042	5180	6943	2884	5147	6879	4990	4195	6620
dF	1			1			1		
F	0.1364			0.3536			0.1968		
*p-value* ANOVA	0.7129			0.5547			0.6613		
% of total variation	0.032			0.2041			0.0475		
*p-value* Time 1 versus Time 2	0.8617	0.9867	0.6314	0.9725	0.4366	0.7802	0.6066	0.0755	0.5755
Cohen’s d	0.03	0.00	0.07	0.01	0.20	0.06	0.06	**0.40**	0.06

	RRi								
	All subjects (N = 27)			Responders (N = 18)			Non-responders (N = 9)		
	Wake	N3	REM	Wake	N3	REM	Wake	N3	REM
Time 1									
Mean	871.3	973.4	922.7	859.3	980.6	917.9	895.2	959.1	932.4
SD	129.1	161.3	129.9	130.9	170.7	144.1	129.6	149.2	103.0
SEM	24.8	31.0	25.0	30.8	40.2	34.0	43.2	49.7	34.3
Range	513.6	721.3	568.3	513.6	688.0	568.3	339.1	410.0	284.0
Time 2									
Mean	870.4	949.0	910.8	854.0	943.9	905.4	903.2	959.2	921.8
SD	136.8	140.8	126.5	144.9	144.7	147.6	119.8	140.7	74.2
SEM	26.3	27.1	24.3	34.2	34.1	34.8	40.0	46.9	24.7
Range	650.9	662.7	607.6	650.9	662.7	607.6	385.8	450.6	242.4
dF	1			1			1		
F	0.7070			0.8325			0.0017		
*p-value* ANOVA	0.4030			0.3658			0.9679		
% of total variation	0.1947			0.3661			0.0012		
*p-value* Time 1 versus Time 2	0.9720	0.3420	0.6431	0.8772	0.2933	0.7179	0.8227	0.9989	0.7681
Cohen’s d	0.01	0.16	0.09	0.04	0.23	0.09	0.06	0.05	0.07

	pNN50								
	All subjects (N = 27)			Responders (N = 18)			Non-responders (N = 9)		
	Wake	N3	REM	Wake	N3	REM	Wake	N3	REM
Time 1									
Mean	31.8	39.0	28.0	25.1	34.1	23.7	45.2	48.7	36.6
SD	22.0	26.5	23.5	19.5	26.3	22.0	21.4	25.4	25.4
SEM	4.2	5.1	4.5	4.6	6.2	5.2	7.1	8.5	8.5
Range	70.6	79.4	72.4	65.8	79.4	72.4	57.2	73.2	66.7
Time 2									
Mean	30.4	28.5	22.6	26.3	25.5	20.4	38.5	34.5	26.9
SD	20.8	23.2	22.7	19.3	22.6	24.7	22.2	24.4	18.6
SEM	4.0	4.5	4.4	4.6	5.3	5.8	7.4	8.1	6.2
Range	62.6	68.6	78.8	62.7	64.6	78.8	53.1	65.4	52.1
dF	1			1			1		
F	5.423			1.280			6.129		
*p-value* ANOVA	**0.0225**			0.2632			**0.0207**		
% of total variation	1.530			0.6238			4.988		
*p-value* Time 1 versus Time 2	0.7446	**0.0169**	0.2095	0.8168	0.1198	0.5444	0.3571	0.0573	0.1891
Cohen’s d	0.07	**0.42**	0.23	0.06	**0.35**	0.14	0.23	**0.63**	**0.40**

Significant values and moderate Cohen's d values are in bold.

When we compared the HRV parameters between responders and non-responders in both periods, the only parameter pNN50 was significantly higher at admission in non-responders at wake phase (*p* = 0.0371, d = 0.98).

We have not found any significant correlation between HRV parameters at Time 1 and scores of any rating scales (MADRS, HAM-A, AIS, CDI), nor of age.

## Discussion

The present study brings new and original insights on autonomic regulation/dysregulation assessed by HRV during sleep in depressed paediatric patients before and after vortioxetine treatment in hospital. To the best of our knowledge, it is a first study describing effects of this antidepressant on HRV. The major findings of this study are following: (1) two thirds of depressed adolescent patients improved their depressive symptoms as well as symptoms of anxiety and insomnia after a short vortioxetine treatment during hospitalization; (2) short vortioxetine treatment significantly decreased parameters pNN50, RMSSD, and HF-HRV during N3 sleep stage in entire group but not in responder group separately. These major findings and possible mechanisms are discussed below.

Applying psychiatric rating scales showed that adolescent patients in our study suffered on average from moderate depression, mild to moderate anxiety and insomnia symptoms such as difficulties with falling asleep, night awakenings, shorter total sleep time, and worsened sleep quality. Vortioxetine proved efficacy, significantly improving the clinical status of depressed paediatric patients within a mean hospital treatment duration of 15.2 days, as evidenced by a 50% decrease in MADRS and CDI scores in two-thirds of the subjects (67%). Currently, pharmacological options for the treatment of depression and anxiety disorders in childhood are limited to fluoxetine in Slovakia, while in the United States, escitalopram is also utilised. Limited studies on the use of vortioxetine for paediatric depression have characterised its pharmacokinetic properties and confirmed its safety but have not shown statistically significant efficacy over placebo^21–23^. Improvement in CDI scores was significant in responder group in all five subcategories (negative mood, interpersonal problems, ineffectiveness, anhedonia, and negative self-esteem) while in no subcategory in non-responder group.

Additionally, our treatment of acute MDD episode significantly improved sleep measured by AIS and anxiety symptomatology measured by HAM-A. In the context of sleep disorders, Du et al. reported a case, in which a three-month trial of vortioxetine at 10 mg led to an improvement in REM sleep behaviour disorder (RBD)^26^. We have also previously described a case report of depressed adolescent that took vortioxetine and improved symptoms of RBD^27^. Insomnia is one of the most common symptoms occurring in depressed patients. The presence of depressive symptoms often results in disruptions to sleep, suggesting that the enhancement in subjective sleep quality scores may stem from a reduction in depressive symptomatology. However, it is also important to mention that the hospitalization of a patient is associated with a regular regimen, which may also contribute to improvement of subjective sleep assessment scores. In adults, vortioxetine at doses 2.5–10 mg once daily did not show significant improvements in HAM-A scores in patients with primary generalized anxiety disorder^28^. We demonstrated a significant reduction of the described anxiety symptomatology in the paediatric patients. Thus, this is the first study describing the clinical effect of vortioxetine on anxiety symptomatology in a population of children and adolescents.

The improvement in the clinical condition of the patients that we demonstrated in this work using scaling methods (MADRS, CDI, HAM-A) can also be interpreted as an improvement of ED, which in the context of polyvagal theory should lead to an increase in vagal activity and HRV. Based on the NIM, we expected an increase in parasympathetic (PNS) activity indicating increased prefrontal activity resulting in inhibition of subcortical regions associated with a reduction in depressive symptomatology.

We have firstly looked at the entire sample group but considering the clinical improvement (50% decrease on MADRS), we further analysed separately the sample of vortioxetine responders and non-responders. The results in the whole group showed a reduction in the vagal parameters pNN50, RMSSD and HF-HRV during N3 sleep stage after vortioxetine treatment compared to the baseline values. However, we have not found any significant effect of vortioxetine only in responder group, even though there was a trend, and the effect size was considered moderate. The reason can be a small sample size and a short duration of the treatment. Since the effect of vortioxetine on HRV has not yet been investigated, it can be discussed in the context of other work on SSRI treatment. The results of these studies, however, have also been inconclusive, showing a non-significant or negative effect of SSRI treatment in adult patients. Specifically, Kemp et al.^15^ did not demonstrate an effect of SSRIs on HRV, whereas Licht et al. demonstrated a reduction in HRV during SSRI use. Patients who started taking SSRIs experienced a reduction in HRV whereas patients who discontinued SSRIs experienced an increase in HRV. The results of Licht et al.^29^ are thus consistent with our results.

In the population of children and adolescents, there have been published only four studies examining the effect of antidepressants on HRV. In two of them, the patients were taking tricyclic antidepressants whose effect on HRV due to their anticholinergic effects cannot be compared with results in our research^30,31^. Vloet et al. in their cross-sectional study on 23 depressed patients did not observe significant effect of antidepressant treatment with SSRIs and mirtazapine on HRV^32^. Koenig et al. demonstrated an increase in HRV associated with an increase in cortical thickness in frontal region after 8 weeks of SSRIs (fluoxetine or sertraline) use, which subsequently may influence HRV due to predicted increase in vagal activity^33^. We hypothesize that one of the reasons for different results may be a difference in the drug chosen and duration of the treatment. It can be assumed that the antidepressant effect associated with the inhibitory effect of the frontal region on subcortical structures correlates with the length of antidepressant treatment (a sufficient period is needed for adaptation of physiological mechanisms regulating autonomic activity).

Interestingly, in the non-responder group, we have observed a significant decrease in parameter RMSSD during N3 sleep stage and in parameter HF during REM sleep. Transition from non-REM to REM sleep stage is characterised by decrease of vagal contribution to HR control, resulting in a relative prevalence of low-frequency HRV, which can be interpreted as a condition of sympathetic (SNS) dominance in the autonomic balance (complex character of LF-HRV: mainly PNS at rest, but reflects SNS dominance in the periods of demand)^25^. Thus, we found the more profound effect of this parasympathetic to sympathetic transition in non-responder MDD patients. However, the variability of this result was very high, which together with the small sample size do not enable us to draw any clear conclusion of this finding and the bigger studies in future should confirm this.

Moreover, HRV measurement can be influenced by several factors that can bias the results. According to Herzig et al. the appropriate measurement time is during deep sleep, which minimalizes confounding factors^34^. Consistently, our study demonstrated that changes in vagal regulation can be captured during N3 stage while they were not detectable during wakefulness and REM phase (apart from the previously mentioned result with high variability). There is the most pronounced (PNS) modulation of cardiac activity during the N3 phase^35^, which probably explains the highest sensitivity of this sleep stage to study changes in PNS activity. Another mechanism may involve the effect of vortioxetine on the *nucleus tractus solitarii* (NTS). Inhibition of serotonin reuptake as well as direct agonism and antagonism of vortioxetine at serotonin receptors interact with cardiovascular regulation at many levels from the PFC to the cardiac receptors^36^. If we assume that clinical improvement in patients correlates with an increase in prefrontal activity, higher inhibitory effect and a subsequent reduction in the activity of subcortical structures, then demonstrated reduction in vagal regulation of the heart may result from a possible effect of vortioxetine on the regulatory centres responsible for cardiovascular integrity. A possible explanation for the reduced cardiovagal activity evaluated by HRV could be precisely the antagonism on 5HT-3 localized on the NTS. Activation of 5HT-3 receptors on the NTS leads to vagal bradycardia whereas their inhibition attenuates the activity of the cardioinhibitory centre and thus leads to tachycardia as a consequence of reduced vagal influences^36^. Therefore, it is not excluded that an increase in prefrontal activity with subsequent subcortical inhibition represents only a secondary effect on vagal-mediated effect on HRV, which is masked by a dominant effect at the NTS level. Considering these assumptions, it is important to make further research on autonomous nervous system and antidepressant therapies using highly selective drugs and longer observation period.

Furthermore, our correlation analysis did not confirm the relationships between the results of the scales assessing depressive symptomatology and HRV parameters during any of the sleep stages. Meta-analysis including healthy adolescent subjects did not find a significant correlation between depressive symptomatology and HRV^33^. There is a greater number of studies addressing this topic in adults in contrast to the paediatric population. Kemp et al. found a correlation between depressive symptomatology and HRV reduction in adult patients in their meta-analysis^15^. According to the latest meta-analysis in children and adolescents, there was no significant association in HRV parameters and depression symptoms, however, gender was a significant moderator for this relationship, revealing a more pronounced negative association among a group with the greater percentage of females^37^. This correlates with our results and percentage of girls in the present study. We have neither demonstrated a significant correlation between HRV and anxiety symptomatology nor sleep disturbances.

This study has several limitations. Hospitalization itself can influence the HRV parameters in both ways—it can represent a stressful uncomfortable situation but at the same time a regular regimen can have a positive effect on sleep and other symptoms. The study included a small sample size, and a relatively short vortioxetine treatment period was analysed, although the clinical improvement was observed. Moreover, there was a gender disparity (more female than male patients) and a lack of a control group. Considering no control nor placebo group in this study, we cannot draw any clear conclusion on the effect of vortioxetine on the clinical psychiatric symptoms in this group of adolescent patients. Future studies with a proper design (randomized placebo-controlled) need to confirm the present pilot findings.

To sum up, in this study we have observed a clinical efficacy of short vortioxetine treatment in two thirds of the hospitalized children and adolescents with depressive disorder. Moreover, improvement of sleep and anxiety symptoms was also noted. This short treatment led to decrease of pNN50, RMSSD and HF-HRV during N3 sleep phase in the entire group but this effect was not confirmed in the responder group separately. We have confirmed that appropriate HRV measurement is during deep sleep minimizing confounding factors. Decreased HRV parameters in this study could be explained by the antagonist effect of vortioxetine on 5HT-3 receptors localized on the NTS but further studies using selective substances and longer observation period are warranted.

## Methods

### Participants

This study included 30 adolescent patients (12–18 years old, 9 boys) with the diagnosis of MDD without psychotic symptoms according to DSM-5^38^ admitted to Psychiatric Clinic of Jessenius Faculty of Medicine and University Hospital Martin in Slovakia. Exclusion criteria were as following: bipolar affective disorder type I and II, autism, substance use disorder in last 6 months, mental anorexia and/or bulimia, intellectual disability (IQ ≤ 70), neurologic disorder, currently on medication significantly affecting sleep architecture, pregnancy, obesity or malnutrition, cardiovascular, endocrine, metabolic, and other diseases affecting the ANS, and alcohol or caffeine intake. Patients were antidepressant-free at least for a month before the inclusion in our study. Regarding their history, 11 individuals had previous experience with antidepressants (fluoxetine, fluvoxamine, or sertraline). The process of final inclusion of the participants is showed in Fig. 1.

The study was approved by the Ethics Committee of Jessenius Faculty of Medicine in Martin (EK87/2019) and the study subjects as well as their parents/legal guardians were informed about the study details and gave written consents to participate.

### Study protocol

At hospital admission, patients’ condition and symptoms were thoroughly assessed by the paediatric psychiatrists and psychiatric rating scales such as Montgomery-Asberg Depression Rating Scale (MADRS), Hamilton Anxiety Rating Scale (HAM-A), Athens Insomnia scale (AIS), and Child Depression Inventory (CDI). Subsequently at night, the first PSG assessment was performed and the next day vortioxetine was administered for the first time. The initial dose was 5 mg a day and was titrated according to the clinical symptoms and response up to maximum dose of 20 mg once daily in the morning (naturalistic setting). The follow-up PSG assessment was made in the end of acute symptom hospitalization (15.2 days on average) and patients were divided into responders (clinical improvement of at least 50% in MADRS; hospital discharge) and non-responders (sent to long-term psychiatric care facilities).

### Clinical assessment

The MADRS is a clinician-rated measure of depressive severity and comprises 10 items that enable to detect depression changes. The items are evaluated on a seven-point Likert scale (0–6), thus the maximum overall score is 60 points. Scores 0–6 represent the absence of symptoms, 7–19 mild depression, 20–34 moderate depression and 35–60 indicate the greatest depression severity^39,40^. Although MADRS is preferably used in adults, its reliability was confirmed also in children^41^. We have used this instrument together with the CDI, a self-report scale, which measures depression symptoms specifically in children and adolescents. It includes 27 items rated 0–2 with a total score up to 54 points. The cut-off score that differentiates between the depressive and general population is 19^42,43^.

The HAM-A is a 14-item rating tool administered by clinicians to measure the severity of anxiety symptoms. Each item is scored from 0 (not present) to 4 (severe). Scores of 17 and less indicate mild symptoms, 18–24 mild to moderate anxiety symptoms and 25–30 moderate to severe anxiety severity^44^. Assessment in adolescent population has been also validated^45^.

The AIS is a valid instrument for insomnia assessment and screening in adolescents. It contains 8 items rated from 0 (no symptoms) to 3 (serious problem). The original cut-off point was 6^46^ while the newer study suggested it to be 7^47^.

These rating scales were administered at admission and at the day of PSG follow-up.

### Polysomnography

PSG analyses were conducted in a specialized room designed to minimize any potential sources of stress. This room was equipped with a camera for monitoring, and the actual assessment took place in an adjacent room. To ensure patients were comfortable and familiar with the surroundings, they were given ample time to acquaint themselves with the room before the analysis began. For ECG monitoring, 7 ECG channels (3 physical and 4 derived) were used as part of the polysomnograph. Three physical electrodes were placed just below the right clavicle (collar bone) and to the left of the midclavicular line (slightly lower than right) and the fifth intercostal space^48^. The sampling rate for ECG monitoring was 500 Hz. ECG was continuously recorded using Sleepware G3 software^49^. Once the electrodes were in place, patients were allowed to rest in the room and naturally fall asleep while being monitored. During the monitoring process, we carefully identified the different sleep stages and recorded the corresponding time periods of electrocardiogram (ECG) activity during both non-rapid eye movement (NREM) and rapid eye movement (REM) sleep phases.

### Heart rate variability assessment

HRV parameters were evaluated from ECG recordings during wakefulness and individual sleep stages (REM and N3 stage of NREM) measured during the regular sleeping time of patients of duration at least 7 h by Alice 6 device (Philips Respironics, Canada) and programme Sleepware G3 before and after short antidepressant therapy during hospitalization (on average 15.2 days). After recording, ECG were manually controlled, and artifacts removed. Then the R-R intervals of 300 time-series long were exported from ECG recordings to csv files and used in the ECG analysis for evaluated time and spectral HRV parameters. Artifacts were detected and removed or replaced automatically according to HRVAS IBI^50^. More specifically, artifacts were detected according to a time-varying threshold (TV-TH) estimated from the time-varying distribution of R-R intervals. First, the quartile deviation of 90 surrounding beats was counted for each beat. Next, the results were multiplied by a factor 5.2. Then, ectopic beats in the form positive–negative-positive or negative–positive-negative, short beats form negative–positive and long beats form positive–negative patterns into the R-R time series were included as artifacts. Subsequently, extra or missed beats were detected by comparing R-R values with median of the surrounding 10 R-R intervals (medianRR). Additionally, an extra beat was detected if the R-R time series condition has been met:

$$\left| {RR\left( i \right) + RR\left( {i + 1} \right) - medianRR\left( i \right)} \right| < 2 * TV - TH,$$

And the missed beat was detected according the condition as follows:

$$\left| {\frac{RR\left( i \right)}{2} - medianRR\left( i \right)} \right| < 2 * TV - TH$$

where RR(i)—representing the R-R interval, RR(i + 1)—characterizing the following R-R interval after RR(i), medianRR – was the median of the surrounding 10 R-R intervals, and TV-TH was a time-varying threshold. Consequently, ectopic beats were corrected by replacing artifact R-R intervals (too long, or too short beats) by interpolation, extra beats were corrected by removing the extra R-oscillation and recalculating the R-R interval series, and missed beats were corrected by adding new R-oscillation occurrence time^50^.

In time-domain analysis, mean R-R intervals as the average of beat-to-beat deviations in the duration of consecutive R-R intervals and the root square of consecutive differences (rMSSD, ms) were assessed independently during the stages of long sleep (N3 and REM) and wakefulness before sleep^51,52^. In spectral power analysis, time series of R-R intervals were resampled using cubic spline interpolation with a frequency of 4 Hz. Subsequently, detrending was performed using the smoothing parameter Λ = 500^50^. Then, spectral powers in the low-frequency band (LF-HRV; 0.04–0.15 Hz) and high frequency band (HF-HRV; 0.15–0.40 Hz) of HRV were analysed using fast Fourier transform with Welch periodogram^52^ during monitoring sleep phases.

Time- and frequency-domain methods of the HRV analysis are the most frequently used for personalised antidepressant treatment^16^. More specifically, time-domain parameters RMSSD and pNN50 represent measures mediated mainly by parasympathetic efferent nerves^53^. Further, frequency (spectral) analysis assesses the underlying variance (power) in the HR pattern at different frequencies. High frequency (HF) is a marker of cardiovagal and emotional modulation, and low frequency (LF) is more complex—it may be produced by both the PNS and SNS, and blood pressure regulation via baroreceptors^51^. The evaluated parameters with their characteristics are displayed in Table 3.

**Table 3:** HRV parameter characteristics.

Parameter	Description	Unit	ANS branch
Time domain			
RRi	Average duration of R-R interval	ms	SNS, PNS
pNN50	Percentage of successive R-R intervals that differ by more than 50 ms	%	PNS
RMSSD	Root mean square of successive R-R interval differences	ms	PNS
Frequency domain			
LF-HRV	Low-frequency power (0.04–0.15 Hz)	ms^2^	baroreflex (SNS, PNS)
HF-HRV	High-frequency power (0.15–0.4 Hz)	ms^2^	PNS

ANS—autonomic nervous system; PNS—parasympathetic nervous system; R-R interval—interval between two R-peaks; SNS—sympathetic nervous system.

### Statistical analyses

The data were visualized and statistically analysed by the GraphPad 8.0.1 (GraphPad Prism, San Diego, CA, USA). Shapiro–Wilk test of normality was performed to assess parametric and non-parametric distribution of the data. The effect of short antidepressant treatment determined via psychiatric rating scale scores was analysed by paired t-test (parametric data) or Wilcoxon test (non-parametric data). Spearman tests were used to assess the correlations between HRV parameters and psychiatric rating scales scores, and age. Mixed ANOVA was used to compare HRV parameters in two times (Time 1, Time 2) and different stages (wakefulness, N3 stage, REM) in three groups (all subjects, responders, non-responders) and then in responders compared to non-responders. The p-value was corrected *post-hoc* by Benjamini, Krieger and Yekutieli two-stage step-up method to control false discovery rate. Such P-value ≤ 0.05 was defined as statistically significant. Effect size was calculated by *post-hoc* comparison of means and standard deviations of two groups using G*Power 3.1.9.4 (Dusseldorf University, Dusseldorf, Germany).

### Ethical approval

The study was approved by the Ethics Committee of Jessenius Faculty of Medicine in Martin (EK87/2019) and all the methods were performed in accordance with the Declaration of Helsinki and other relevant guidelines and regulations.

### Informed consent

To ensure ethical standards are upheld, all participants involved in this study and their parents/legal guardians have been provided with comprehensive information regarding the research objectives, procedures, potential risks, and benefits. Furthermore, each participant has voluntarily agreed to participate by providing explicit informed consent. Informed consent was obtained also from their parents/legal guardians.

## Data Availability

The datasets used and/or analysed during the current study are available from the corresponding author on request.
